# Biochar Decreases Fertilizer Leaching and Promotes Miscanthus Growth in Saline-Alkaline Soil

**DOI:** 10.3390/plants12203649

**Published:** 2023-10-23

**Authors:** Manlin Xu, Qiqi Sun, Qiangbo Liu, Guo He, Congpeng Wang, Kang He

**Affiliations:** 1 Shandong Peanut Research Institute, Qingdao 266100, China; xumanlin@126.com (M.X.); sunshine19890707@163.com (Q.S.); 2 National Key Laboratory of Wheat Improvement, College of Life Sciences, Shandong Agricultural University, Tai’an 271018, China; liuqiangbo@sdau.edu.cn; 3 Qingdao Institute of Bioenergy and Bioprocess Technology, Chinese Academy of Sciences, Qingdao 266100, China; heguo@qibebt.ac.cn; 4 College of Landscape Architecture and Forestry, Qingdao Agricultural University, Qingdao 266109, China; wangcongpeng@qau.edu.cn

**Keywords:** biochar, fertilizer leaching, soil depth, enzyme activity, saline-alkaline soil

## Abstract

Biochar has been widely reported to improve soil conditions and affect plant growth. However, its effectiveness is limited by soil type and production technology. Considering the application effect of biochar in saline alkali soil, there is currently a lack of in-depth mechanism explanations in the research. Therefore, we designed an experiment to explore the effect of biochar on plant growth in saline alkali soil and conducted soil column experiments in a greenhouse environment using composite inorganic fertilizer (NPK). The results showed that biochar significantly affected the distribution of soil nutrient content at different depths, with a significant increase in fertility levels in the surface and middle layers and a decrease in fertility levels in deep soils. Compared to using fertilizers alone, the combined use of biochar and fertilizers further expands the enrichment effect and significantly reduces the leaching of fertilizers into deeper layers. At the same time, the application of biochar also improved soil properties, including an increase in electrical conductivity and organic matter content, as well as an increase in soil enzyme activity. On the other hand, the application of biochar also increases the activity of antioxidant enzymes and the content of osmoregulation substances in plants, reducing the environmental stress that plants are subjected to. Therefore, our results indicate that biochar can reduce the leaching of fertilizers into deep soil layers, improve soil properties, and promotes the growth of Miscanthus in saline alkali soils.

## 1. Introduction

Biochar is a solid carbon-rich material that is produced through pyrolysis under limited oxygen conditions and contains metal elements. It can be used directly as a soil conditioner and fertilizer, thus exerting positive effects on soil health and crop growth [1,2]. Natural biochar is rich in elements such as P, K, Ca, and Mg [3,4]. Because of their pore structures, biochars (such as orange peel biochar) have excellent adsorption as catalyst-support materials [5]. The performance of several types of biochars in the adsorption of inorganic and organic contaminants from aqueous solution has been reported [6,7]. The combination of biochar and fertilizers improves the long-term release effect of fertilizers; a possible explanation is that the porous nature of biochar can slowly release fertilizers through adsorption, while improving soil properties can enhance fertilizer availability and achieve long-term effectiveness [8,9]. Biochar fabricated from magnesium-rich tomato tissues has been found to adsorb a substantial amount of phosphorus (>100 mg 106 g^−1^), thus confirming the potential of some phosphorus-laden types of biochar to serve as slow-release fertilizers [10]. Similarly, cow dung biochar with high phosphorus adsorption abilities has also been found to enhance the growth of lettuce [11].

Inorganic fertilizers are more conventional and more commonly used than biochar. They play major roles in increasing crop yields and alleviating the food crisis; however, their irrational long-term use has led to severe agricultural and environmental problems [12]. For instance, the leaching (NO_3_^−^), volatilization (NH_3_^+^), and denitrification (N_2_ and N_2_O) of nitrogen fertilizers causes a low N use efficiency by crop plants, degradation of water quality, and ecosystem disruption. The low utilization of nitrogen fertilizer in crop production can be affected by soil properties, crop absorption rates, and other factors. A major reason for fertilizer loss is the lack of synchronization between the release of nitrogen in fertilizers and the nitrogen demand of the crop [13]. Achieving ideal fertilizer management models is challenging, owing to the complex processes of soil fertilizer decomposition, metabolism, and cycling. Therefore, a strategy to enhance the long-term release of fertilizers to improve the crop utilization efficiency of fertilizers, while decreasing fertilizer leaching and improving soil health and environmental safety, is critical for increasing crop yields [14,15,16].

Plant roots are the main functional organization for plants to absorb nutrients from soil. Biochar has been reported to promote the growth of crop roots, as indicated by increased root biomass [17]. For instance, biochar is known to modify soil properties (such as pH, nutrient availability, aeration, or water-holding capacity), thereby affecting root growth [18]. Changes in soil microorganisms and the release of root exudates caused by biochar also directly or indirectly induce a series of changes in the root growth environment, thus affecting the growth status of plants [19,20]. Biochar also affects the absorption and utilization of fertilizers, such as the significant enrichment of nitrogen fertilizer in wheat rhizosphere soil [21,22].

The properties of biochars can differ markedly because of the diverse processes and resources involved in its production. In addition, the soil type influences the effects of biochar on plant growth [23]. Our study was aimed at investigating whether biochar can reduce fertilizer leaching in saline alkali soils and promote plant growth in saline alkali soils. We used saline alkali soil for plant growth because the loss of fertilizer in saline alkali soil is relatively severe [24]. We hypothesized that the addition of biochar would reduce the loss of fertilizer to the deep soil layer and improve the enrichment of nutrients (Hypothesis 1); and the biochar would improve soil properties and promote plant growth (Hypothesis 2).

## 2. Results

### 2.1. Biochar Affects Soil Fertility Properties

**Figure 1:**
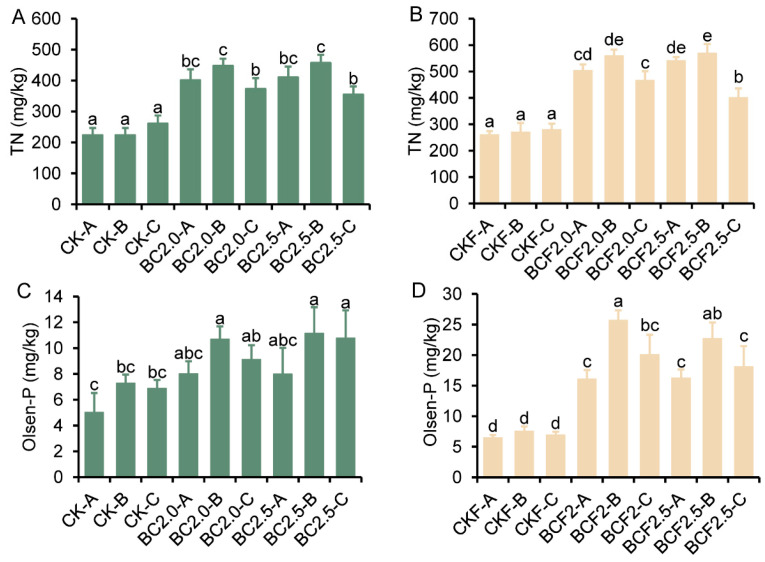
The content of total nitrogen and phosphorus in soil under different treatments. (**A**–**D**) represent the TN and Olsen-P contents in different soil depth layers under Control (CK), 2.0% biochar (BC2.0), 2.5% biochar (BC2.5), fertilizer (CKF), 2.0% biochar plus fertilizer (BCF2.0), and 2.5% biochar plus fertilizer (BCF2.5) treatments. -A represents 0~30 cm, -B represents 30~60 cm, -C represents 60~90 cm. Different letters indicate significant differences from the control by the one-way analysis of variance (ANOVA) and Duncan’s multiple range test (*p* < 0.05).

The treatments involving biochar alone (BC2.0 and BC2.5) and CK resulted in an increase in total nitrogen content (TN) in the soil across various depths. Among the different soil depths, layer B had the highest nitrogen content, followed by layer A, whereas layer C had the lowest nitrogen content. Similar trends were observed in the fertilizer group (CKF, BCF2.0, and BCF2.5). Furthermore, the biochar–fertilizer treatments resulted in a higher soil phosphorus content than observed after treatment with fertilizer alone (CKF) or biochar alone (BC). This finding was observed with both of the compounded biochar fertilizer treatments (Figure 1C,D). These results indicated that biochar improved the retention of fertilizers in the A, B layers.

**Figure 2:**
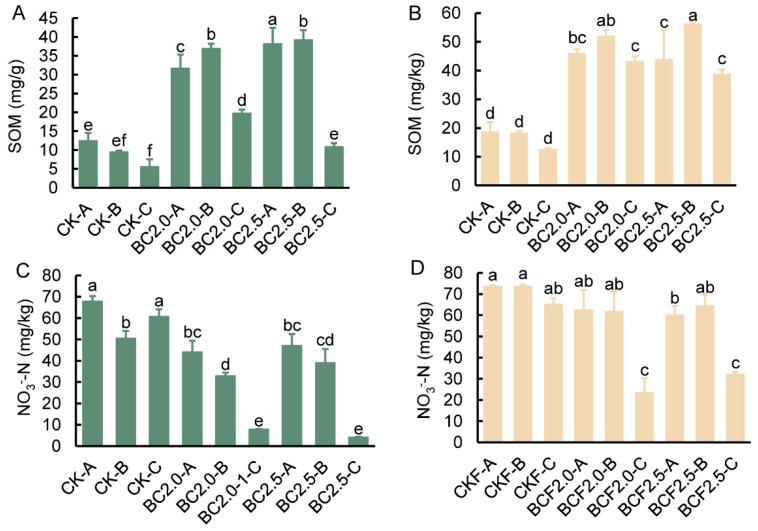
The content of SOM and NO_3_^−^-N in soil under different treatments. (**A**–**D**) represent the SOM and NO_3_^−^-N contents in different soil depth layers under Control (CK), 2.0% biochar (BC2.0), 2.5% biochar (BC2.5), fertilizer (CKF), 2.0% biochar plus fertilizer (BCF2.0), and 2.5% biochar plus fertilizer (BCF2.5) treatments. -A represents 0~30 cm, -B represents 30~60 cm, -C represents 60~90 cm. Different letters indicate significant differences from the control by the one-way analysis of variance (ANOVA) and Duncan’s multiple range test (*p* < 0.05).

The addition of biochar led to a consistent increase in SOM; the highest content was observed in the B layers, and the lowest content was observed in the C layer (Figure 2A,B). SOM significantly decreased with increasing soil depth. After fertilizer treatment, the overall SOM trend remained B > A > C, owing to the addition of exogenous fertilizers; however, the decrease in the C layer was not significant. Interestingly, under CK and CKF treatments, the SOM content was A > B > C, thus indicating that the biochar significantly improved the SOM of B layer. Contrary to our expectations, the addition of biochar significantly decreased the content of nitrate nitrogen, thus resulting in a nearly five-fold decrease in the C layer across depth levels (Figure 2C,D). In the fertilizer group, the nitrate nitrogen content also decreased with depth, whereas the addition of biochar enhanced this trend from unapparent to significant.

### 2.2. Biochar Affects Soil EC and Water Content

**Figure 3:**
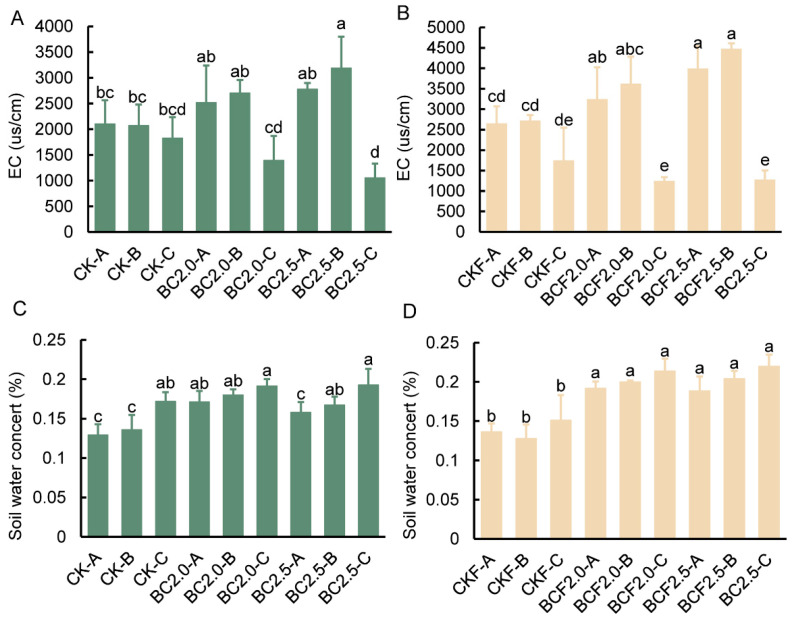
The content of EC and soil water content in soil under different treatments. (**A**–**D**) represent the EC and soil water content contents in different soil depth layers under Control (CK), 2.0% biochar (BC2.0), 2.5% biochar (BC2.5), fertilizer (CKF), 2.0% biochar plus fertilizer (BCF2.0), and 2.5% biochar plus fertilizer (BCF2.5) treatments. -A represents 0~30 cm, -B represents 30~60 cm, -C represents 60~90 cm. Different letters indicate significant differences from the control by the one-way analysis of variance (ANOVA) and Duncan’s multiple range test (*p* < 0.05).

The addition of biochar significantly increased the soil EC in the A, B layers, and the B layer showed the highest increase, from 30.4% to 53.9% (Figure 3A). In the fertilizer group, this increasing trend was further maintained, resulting in a significant improvement in layers A and B (33.2% and 64.7%, respectively) compared with CKF (Figure 3B).

The soil water content increased overall in all soil depth layers after biochar treatment. Interestingly, water content showed an opposite trend from that of EC: treatments containing biochar had a higher soil water content (Figure 3C,D). The results indicated that biochar also enhanced water absorption in the A,B layers compared with CK and CKF.

### 2.3. Biochar Affects the Distribution of Soil Ions

**Figure 4:**
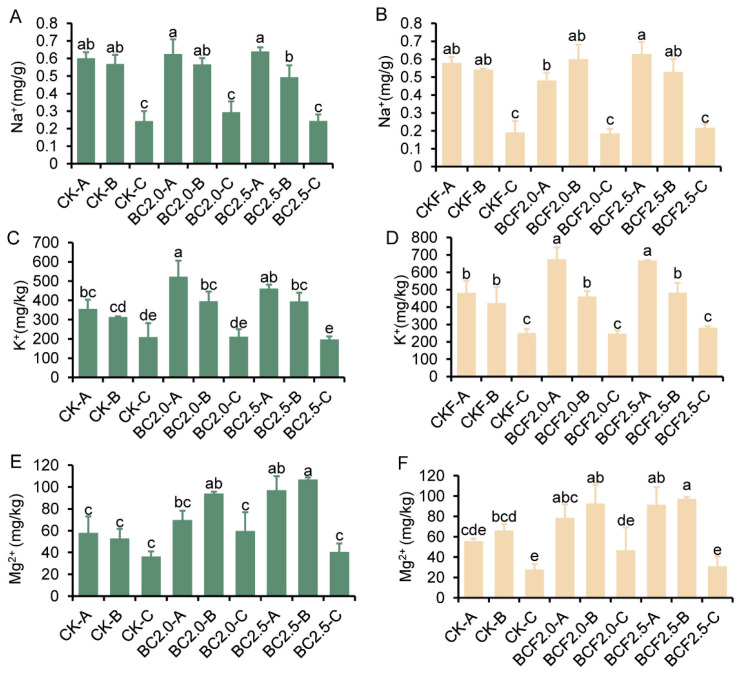
The content of soil ions under different treatments. (**A**–**F**) represent the Na^+^, K^+^, Mg^+^ contents in different soil depth layers under Control (CK), 2.0% biochar (BC2.0), 2.5% biochar (BC2.5), fertilizer (CKF), 2.0% biochar plus fertilizer (BCF2.0), and 2.5% biochar plus fertilizer (BCF2.5) treatments. -A represents 0~30 cm, -B represents 30~60 cm, -C represents 60~90 cm. Different letters indicate significant differences from the control by the one-way analysis of variance (ANOVA) and Duncan’s multiple range test (*p* < 0.05).

The effects on soil ion distribution intuitively indicated the available ion content of different soil layers (Figure 4). The A, B layers showed similar trends of enrichment in potassium (K) and sodium (Na) ions, whereas fewer ions were observed in the C layer. The addition of biochar, compared with the CK treatment, did not lead to significant changes in sodium ions (Figure 4A,B). However, potassium ions significantly increased under the biochar treatments, particularly in the A layer, which increased from 29.8% to 40.7% compared with CK and CKF (Figure 4C,D), respectively. Magnesium (Mg) ions exhibited the same trend as potassium ions, with enrichment in the A, B layers (Figure 4E,F).

### 2.4. Biochar Affect Soil Enzyme Activity

**Figure 5:**
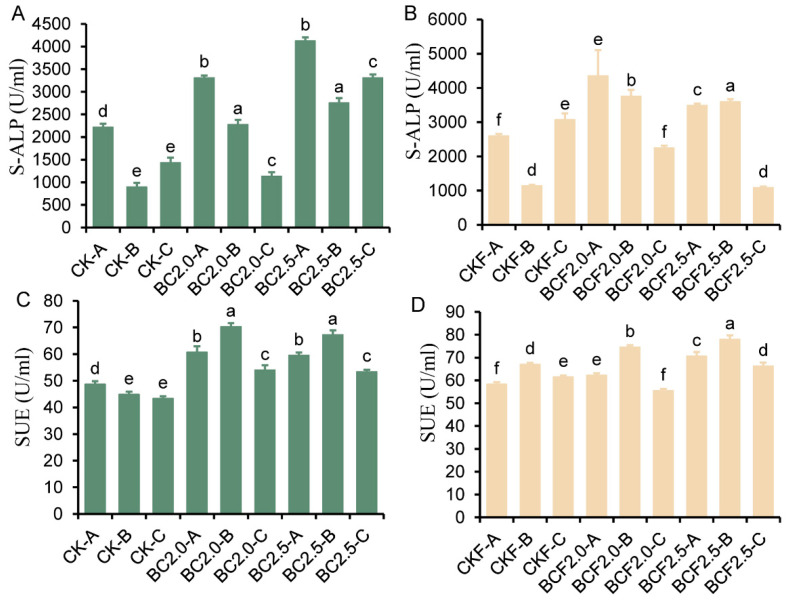
The content of soil enzyme activities under different treatments. (**A**–**D**) represent the S-ALP and SUE contents in different soil depth layers under Control (CK), 2.0% biochar (BC2.0), 2.5% biochar (BC2.5), fertilizer (CKF), 2.0% biochar plus fertilizer (BCF2.0), and 2.5% biochar plus fertilizer (BCF2.5) treatments. -A represents 0~30 cm, -B represents 30~60 cm, -C represents 60~90 cm. Different letters indicate significant differences from the control by the one-way analysis of variance (ANOVA) and Duncan’s multiple range test (*p* < 0.05).

Soil enzymes play important roles in nutrient metabolism and utilization. To further understand the effects of biochar, we measured the activity of two important soil enzymes (Figure 5). The results indicated a significant increase in S-ALP (sulfatase enzyme activity) with the biochar treatments, particularly in the A, B layers. The increase was further enhanced in the BCF groups (Figure 5A,B). The highest increase in S-ALK activity was observed in BCF2.0-A (230% more activity than that in the CKF treatment). Nevertheless, the SUE showed potential enrichment in the B layer of the soil (Figure 5C,D).

### 2.5. Biochar Affects Plant Performance

**Figure 6:**
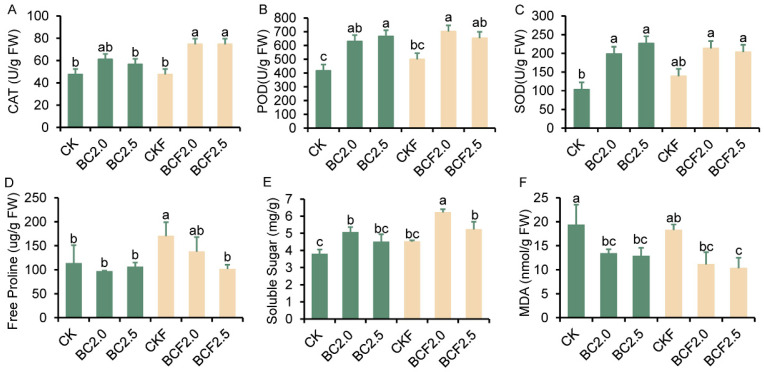
The content of antioxidant enzyme activities of miscanthus under different treatments. Enzyme activities under Control (CK), 2.0% biochar (BC2.0), 2.5% biochar (BC2.5), fertilizer (CKF), 2.0% biochar plus fertilizer (BCF2.0), and 2.5% biochar plus fertilizer (BCF2.5) treatments. Different letters indicate significant differences from the control by the one-way analysis of variance (ANOVA) and Duncan’s multiple range test (*p* < 0.05).

To understand the direct effects of changes in soil properties on plant growth, we measured the content of physiological substances associated with plant stress (Figure 6). We observed a significant increase in the content of peroxidase scavenging enzymes, such as catalase, peroxidase, and superoxide dismutase, after all treatments. Furthermore, the addition of fertilizer combined with biochar resulted in the highest increase in enzyme activity (Figure 6A–C). Additionally, the content of osmoregulation substances, such as free proline (Figure 6D) and soluble sugars (Figure 6E), also showed a significant increase under the biochar treatments. In contrast, the content of MDA (Figure 6F), a marker of oxidative stress, decreased significantly under the biochar treatments. The greatest decrease in MDA content was observed with BCF treatment (a decrease of 42.5% to 44.6% with respect to the CK treatment).

**Figure 7:**
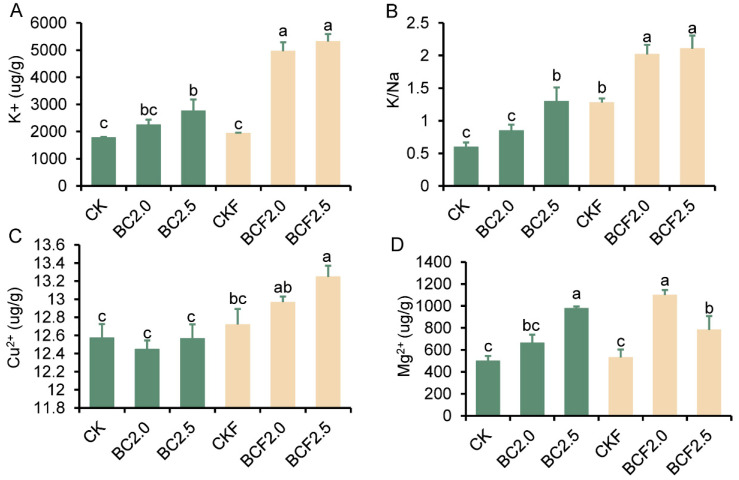
The content of ions of miscanthus under different treatments. Under Control (CK), 2.0% biochar (BC2.0), 2.5% biochar (BC2.5), fertilizer (CKF), 2.0% biochar plus fertilizer (BCF2.0), and 2.5% biochar plus fertilizer (BCF2.5) treatments. Different letters indicate significant differences from the control by the one-way analysis of variance (ANOVA) and Duncan’s multiple range test (*p* < 0.05).

The content of Ions within plants was significantly affected by the applied biochar treatments (Figure 7). Specifically, the content of potassium (K) ions was significantly greater after all biochar treatments than the CK treatment, and the greatest increase was observed after the BCF treatment (Figure 7A). However, applied fertilization alone did not result in an increase in K ion content in the leaves. Additionally, the potassium sodium (K/Na) ratio peaked after the fertilizer treatments, and a rising trend was observed after the BCF and CKF treatments (Figure 7B). The content of copper (Cu) ions and magnesium (Mg) ions followed a similar trend: significant increases were observed after the CKF treatment (Figure 7C,D). Among these treatments, the BCF treatment resulted in the greatest increase in Cu and Mg ion content. These results indicated that added biochar affected the distribution of soil ions, particularly in combination with fertilizer treatments.

## 3. Discussion

### 3.1. Biochar Affects the Distribution of Nitrogen and Phosphorus Fertilizers at Different Soil Depths

Nitrogen and phosphorus fertilizers are widely used in agriculture. However, the utilization rate of these fertilizers is significantly influenced by the unique soil properties of saline alkali soil. The incorporation of biochar has been observed to affect root growth, but this effect depends on the soil type and biochar characteristics [25,26]. Our results showed the significant influence of biochar application on the distribution of nitrogen and phosphorus in the soil across various depths, particularly in the fertilizer group. The application of biochar notably increased the total nitrogen content in the A,B layers, and the B layer showed the highest concentration (Figure 1). These results suggested that biochar mitigated the leaching losses of nitrogen fertilizer in saline alkali soil, potentially because of its direct adsorption capability. In addition, the remediation effects of biochar on saline alkali soil properties, such as enhancing porosity, play a role in decreasing fertilizer loss by changing the soil pH [27,28], For example, in saline alkali soil, nitrogen fertilizer is mainly volatilized in the form of ammonia, and the addition of biochar affects the soil pH, thereby significantly reducing the loss of nitrogen fertilizer [9,29]. Similarly, previous studies also indicated that amending biochar and fertilizer lowers leaching, improves the root–fertilizer contact, and, thus, optimizes the availability of P to plants [8,30].

Beyond the adsorption effect, the higher nutrient content in layer B might be associated with the root absorption effect. Others also reported that the adsorption of biochar and the plant rhizosphere have complementary effects, possible mechanisms may explain these effects: (i) biochar contains several organic compounds (e.g., alkanoic acid, hydroxyl and ethoxy acids) known to stimulate plant root elongation, thus strengthening the adsorption range of nutrients [31,32], and (ii) biochar addition decreases soil mechanical resistance, promoting fine root elongation, and also strengthening the enrichment of nutrients [32,33]. The prominent distribution of available phosphorus in the B layer significantly differs from that in the A,C layers, thus indicating that biochar has direct effects on enrichment in available phosphorus [3,34].

The SOM was significantly associated with the input of organic carbon. The high-carbon content of biochar itself directly contributes to the soil carbon pool and subsequently enhances the organic matter content in the soil. We observed a significant increase in the A, B layers (Figure 2A,B), which might have been associated with the direct or indirect roles of biochar in the soil carbon pool [35]. In contrast, root litter can serve as a direct source of SOM, thus explaining the highest content of SOM observed in the A, B layers [36]. At the same time, there are also reports that roots can accelerate the release of organic matter from biochar, thereby further increasing the soil’s organic matter content [37]. This indicates that biochar can further amplify the modification of the root environment, thereby increasing the organic matter content in the soil [38,39].

In contrast, the application of biochar resulted in lower nitrate nitrogen content than observed in the control conditions. Furthermore, analysis of the nitrate nitrogen distribution across different soil depth levels revealed higher concentrations in the A,B layers than in the C layer (Figure 2C,D). On the contrary, there are reports that the use of biochar reduces the loss of nitrate nitrogen and increases its content in soil, which is explained as the intrinsic properties of biochar, such as its negatively charged surface area, porous structure, and strong ion exchange capacity [40].

The different results in our experiment might be attributable to the selective absorption preferences of both plants and biochar, which have a high preference for nitrate. The observed decrease in nitrate nitrogen content might potentially be explained by the uptake of nitrogen by plant growth, thus leading to lower amounts than observed in the control treatments [29,41].

### 3.2. Biochar Affects the Distribution of Soil Ions across Different Depths

The EC of soil directly reflects the mobility of ions within the soil. Our experiments consistently showed higher EC values in the A, B layers than in the C layer across all treatments (Figure 3A,B). This trend was also observed with the control treatments, CK and CKF, and was probably associated with soil transpiration and rhizosphere adsorption by plants [42]. In contrast, the BC and BCF groups showed a more pronounced trend, with significantly higher EC values in the A,B layers than the C layer. Among treatments, BC2.5 and BCF2.5 exhibited the highest EC values in the B layer. This finding indicated that the biochar promoted the accumulation of nutrient ions and decreased the leaching of nutrient ions to the deep layer [43]. Furthermore, both sodium ions and potassium ions showed enrichment in the A, B layers (Figure 4A–D); the sodium ion content in the A,B layers was not significantly greater after the BC and BCF treatments than the CK treatment, thus indicating that biochar might not induce additional sodium enrichment. K and Mg increased significantly in the A,B layers (Figure 4E,F), thereby suggesting the ability of biochar to enrich these ions. This is attributed to the metal elements inherent in biochar; on the other hand, biochar can provide better buffering capacity to the soil, including nutrient absorption, migration, and release [44]. The variation in ion adsorption by biochar may be associated with improved plant resistance to salt stress [29,45]. Meanwhile, the biochar could release nutrients such as Ca, Mg, K, N, and P to strengthen the offset impact of salt. And, increasing the K concentration in soils to counteract the adverse impacts of Na is one of the major benefits [4]. The distribution of soil enzyme activity also indirectly verified the distribution of nutrient ions (Figure 5A–D). Higher soil enzyme activities have been reported to enhance the decomposition and mineralization of soil organic matter and, hence, nutrient cycling [46]. The significant enrichment in S-ALK and S-UE in the A, B layers indicated the potential of biochar to aid in the adsorption of soil nutrient ions and improve soil microbial indicators [47,48].

### 3.3. Biochar Affects Plant Stress Resistance

The effects of biochar on improving soil properties directly influence the plants’ growth environments, thereby affecting their resistance to saline alkali stress, including increasing the content of osmoregulation substances and promoting stress relative to enzyme activity [4,49]. Salt stress is often accompanied by osmotic stress, which produces a large amount of active free radicals. Consequently, the activity of three representative antioxidant enzymes significantly increased after BC and BCF treatments (Figure 6A–C). In addition, the content of free proline and soluble sugar, two major substances regulating osmotic stress in plants [50], also significantly increased after BC and BCF treatments (Figure 6D,E). On the contrary, the content of MDA, which represents the degree of leaf damage, significantly decreased after the BC and BCF treatments, and the most significant decrease was observed with the BCF treatment (Figure 6F). This indicates that the use of biochar enhances plant stress resistance by increasing antioxidant enzyme activity and the substance content of osmoregulation. Similarly, the potassium/sodium ratio is an important indicator of plant resistance to osmotic stress, and a high potassium/sodium ratio can help plants reduce sodium ion toxicity and drought stress [4,27,51]. In the results, the potassium ions and potassium/sodium ratio in the leaves exhibited a similar increasing trend, and the highest values were observed in the BCF group (Figure 7). Likewise, copper ions and magnesium ions showed similar change trends [52]. These consistent change trends in important ions may contribute to promoting plant growth under adverse conditions (Table S1).

## 4. Methods and Materials

### 4.1. Materials

Biochar was produced from a mixed lignocellulosic biomass composed of pine, poplar, and *Caragana intermedia* (*w*/*w*/*w*, 2:2:1). The mixed lignocellulose was treated with sulfuric acid at 170 °C for 1 h by steam heating and then heated at 300 °C or 2 h. The resulting product was then sieved to a particle size of 2 mm and was then ready for use. The biochar’s basic characteristics were as follows: pH 4.6; TN 4.1 g/kg; TP 0.4 mg/kg; CEC 17.0 coml/kg, and C content 79%. Saline alkali soil samples were collected from Changyi County (119°39′ N, 37°03′ E), Shandong Province. Topsoil samples (0–20 cm) were collected with an S-shaped sampling method. The soil was air dried and sieved through a 2.0 mm mesh to remove large impurities. The basic soil characteristics were as follows: pH 8.9, TN 0.5 g/kg, TP 670 mg/kg, and soluble salt content 8.0 g/kg.

### 4.2. Experimental Design

Soil column tests were conducted in a greenhouse at 26 °C with a 16 h light/8 h dark photoperiod, and with the relative humidity kept at 60–70%. The prepared biochar was mixed with saline alkali soil at concentrations of 2.0% and 2.5%, and then used to fill soil columns (polyethylene plastic soil column, 100 cm long, and 25 cm in diameter) to 30~60 cm depth. The original soil was used for 0~30 cm and 60~90 cm. In fertilizer treatment, inorganic composite fertilizers (N + K_2_O + P_2_O_5_ + TE:30–30–30) provided by Biotechnology Co., (Qingdao, China) were uniformly added at 216 kg N ha^−1^, 216 kg P_2_O_5_ ha^−1^, and 216 kg K_2_O ha^−1^, respectively. Six treatments were used: (i) no amendments (CK); (ii) 2.0% biochar (BC2.0); (iii) 2.5% biochar (BC2.5); (iv) inorganic fertilizer (CKF); (v) 2.0% biochar + fertilizer (BCF2.0); and (vi) 2.5% biochar + fertilizer (BCF2.5). These treatments were further divided into two subgroups: a biochar group (BC2.0 and BC2.5) and a biochar plus fertilizer group (BCF2.0, and BCF2.5). *Miscanthus* sp. plants were selected and colonized with tuber cuttings because it is a valuable energy plant with strong environmental adaptability. Each treatment was administered in three replicates, and full irrigation was performed after transplantation. All soil columns use bottom water supply, which means placing a water tray under each soil column to maintain sufficient water supply. Soil samples and plant samples were collected for measurement after 90 days of growth.

### 4.3. Characterization

The determination of soil indicators was performed according to traditional analytical methods. Soil pH (soil: water, 1:5, *w*/*v*) and electrical conductivity (EC) were measured with a pH meter (PB-10, Sartorius, Germany) and EC meter (FE30-K Plus, Mettler Toledo, USA), respectively. Soil organic matter (SOM) content was determined with the potassium dichromate oxidation method [4]. The soil total soluble salt was extracted with water (soil: water, 1:5, *w*/*v*), and the soil salt content was quantified after air drying. The elemental content was determined with an elemental analyzer (Vario EL III, Elementar, Germany) [4]. The cation exchange capacity of soil and biochar was determined through extraction with 1.0 M ammonium acetate and titration with 50 mM hydrochloric acid. The exchangeable sodium percentage was calculated as the ratio of exchangeable Na^+^ to cation exchange capacity. The content of ammonium N (NH_4_^+^–N) and nitrate N (NO_3_^−^–N) was determined with colorimetric assays after extraction with 0.5 M K_2_SO_4_ (1:5, *w*/*v*). Na^+^, K^+^, Ca^2+^, and Mg^2+^ content was determined with inductively coupled plasma mass spectrometry (MARS5) after microwave digestion [53].

The proline content of plants was determined with a commercial detection kit (A107) provided by Jiancheng Co., (Nanjing, China). The content of malondialdehyde (MDA) was determined with a commercial detection kit (BC0020) provided by Jiancheng Co., (Nanjing, China). To detect the enzymatic activities of superoxide dismutase, peroxidase, and catalase in leaves, we used three detection kits (BC0175, BC0095, and BC0205, respectively) provided by Jiancheng Co., (Nanjing, China) [54]. The Na^+^ and K^+^ content in leaf saps was quantified with a flame atomic absorption spectrophotometer (240 AA; Agilent, Santa Clara, CA, USA) [55].

### 4.4. Statistical Analysis

We analyzed the data in SPSS 17.0 software. Treatments were compared with one-way analysis of variance and Duncan’s multiple range test. Unless otherwise stated, the significance threshold was a *p*-value less than 0.05.

## 5. Conclusions

In response to reports of biochar adsorption on fertilizer, we designed and conducted a targeted soil column experiment to investigate the effects of biochar with different depths of application on soil properties. Our results show that the use of biochar can indeed enhance the retention effect of nutrients in the soil. In particular, compared to individual fertilization, the combined use of biochar and fertilizer significantly reduces the leaching of fertilizer into the deep layer and enhances its retention in the middle layer of the soil. This indicates that applying biochar in the middle layer also has a good fertilizer adsorption effect.

Furthermore, biochar positively influenced key physical and chemical properties of the soil, including enhancement of the activity of alkaline phosphatase and other enzymes. The interactions also promoted the growth of plant roots, thus increasing plant resistance to saline alkali stress and ultimately resulting in a higher growth of miscanthus in saline alkali soil.

Our experiments were conducted in a greenhouse setting and therefore serve as a preliminary study to guide future research. Further empirical validation through field research is needed to better support the use of biochar in agriculture in the future.

## Data Availability

The original contributions presented in the study are included in the article material. Further inquiries can be directed to the corresponding author.

## Supplementary Materials

The following supporting information can be downloaded at: https://www.mdpi.com/article/10.3390/plants12203649/s1.
