# Supplementary material for: Biochar Decreases Fertilizer Leaching and Promotes Miscanthus Growth in Saline-Alkaline Soil
Source: Plants (Basel). 2023 Oct 23;12(20):3649. doi: 10.3390/plants12203649 (PMC10609680; doi:10.3390/plants12203649)
Supplement: Supplementary file 1 [file plants-12-03649-s001.zip › plants-2652058-supplementary.pdf]

Supplementary materials

Supplement Table S1. The Shoot and Root length of miscanthus under different treatments.

	CK	BC2.0	BC2.5	CKF	BCF2.0	BCF2.5
Shoot Length (cm)	33.93	103.93	95.30	48.30	128.80	127.93
Root Length (cm)	11.50	42.40	41.27	13.43	52.43	51.00
